# Brain-Derived Neurotrophic Factor (BDNF)-Induced Tropomyosin-Related Kinase B (Trk B) Signaling Is a Potential Therapeutic Target for Peritoneal Carcinomatosis Arising from Colorectal Cancer

**DOI:** 10.1371/journal.pone.0096410

**Published:** 2014-05-06

**Authors:** Koji Tanaka, Yoshinaga Okugawa, Yuji Toiyama, Yasuhiro Inoue, Susumu Saigusa, Mikio Kawamura, Toshimitsu Araki, Keiichi Uchida, Yasuhiko Mohri, Masato Kusunoki

**Affiliations:** Department of Gastrointestinal and Pediatric Surgery, Mie University Graduate School of Medicine, Tsu, Mie, Japan; Ohio State University Comprehensive Cancer Center, United States of America

## Abstract

Tropomyosin-related receptor kinase B (TrkB) signaling, stimulated by brain-derived neurotrophic factor (BDNF) ligand, promotes tumor progression, and is related to the poor prognosis of various malignancies. We sought to examine the clinical relevance of BDNF/TrkB expression in colorectal cancer (CRC) tissues, its prognostic value for CRC patients, and its therapeutic potential *in vitro* and *in vivo*. Two hundred and twenty-three CRC patient specimens were used to determine both BDNF and TrkB mRNA levels. The expression of these proteins in their primary and metastatic tumors was investigated by immunohistochemistry. CRC cell lines and recombinant BDNF and K252a (a selective pharmacological pan-Trk inhibitor) were used for *in vitro* cell viability, migration, invasion, anoikis resistance and *in vivo* peritoneal metastasis assays. Tissue BDNF mRNA was associated with liver and peritoneal metastasis. Tissue TrkB mRNA was also associated with lymph node metastasis. The co-expression of BDNF and TrkB was associated with liver and peritoneal metastasis. Patients with higher BDNF, TrkB, and co-expression of BDNF and TrkB had a significantly poor prognosis. BDNF increased tumor cell viability, migration, invasion and inhibited anoikis in the TrkB-expressing CRC cell lines. These effects were suppressed by K252a. In mice injected with DLD1 co-expressing BDNF and TrkB, and subsequently treated with K252a, peritoneal metastatic nodules was found to be reduced, as compared with control mice. BDNF/TrkB signaling may thus be a potential target for treating peritoneal carcinomatosis arising from colorectal cancer.

## Introduction

The outstanding progress in the treatment of metastatic colorectal cancer (mCRC) has been founded on the recent multi-drug combination chemotherapies, which now produce median survival times exceeding 20 months [1]. However, peritoneal carcinomatosis (PC) can arise from CRC. PC is associated with extremely poor survival, and very few therapeutic or palliative treatments are available [2]. Thus, a better understanding of the molecular and biological behaviors of the PC arising from CRC is urgently required to facilitate the development of new therapeutic strategies.

Brain-derived neurotrophic factor (BDNF) is a member of the neurotrophin (NT) family. BDNF plays an important role in the development and repair of the nervous system [3]. It binds to its two major receptors, the tropomyosin-related receptor kinase B (TrkB) with high affinity and specificity, and the pan-NT receptor p75 (p75^NTR^) with low affinity [4]. Binding of BDNF to TrkB leads to autophosphorylation of tyrosines in the intracellular domain with activation of downstream signaling pathways such as RAS/MAPK and PI3K/AKT [4], [5]. BDNF also binds low affinity receptor p75^NTR^ which exerts diverse functions such as the regulation of cell survival and differentiation during neuronal development [6].

Although both TrkB and p75^NTR^ involved in proliferation, differentiation, survival and apoptosis of neuronal and non-neuronal tumors [7], [8], p75^NTR^ preferentially acts as an interacting partner of TrkB, modulates TrkB activation by BDNF, and influences prosurvival effect by BDNF/TrkB signaling [9].

BDNF/TrkB signaling has been reported to be associated with tumor progression, metastasis, and response to chemotherapy in several human malignancies such as neuroblastoma [10], ovarian [11], head and neck [12], lung [13], hepatocellular [14], pancreatic [15], bladder [16], prostate [17], multiple myeloma [18], and breast tumor [19]. TrkB has also been shown to promote resistance to anoikis (a form of detachment-induced apoptosis) [20], and thereby to confer metastatic properties or epithelial-mesenchymal transition (EMT) [21], [22]. In contrast to the role of TrkB in cancer, p75^NTR^ seems to have either tumor-promoting or tumor-suppressing functions according to tumor types [8].

Previously, studies in our laboratory have revealed: an association between TrkB levels tumor progression and patient prognosis in gastric cancer [23]; the association of TrkB with chemotherapy resistance in esophageal cancer [24]; and TrkB's involvement in the EMT of colorectal cancer [25]. More recently, we have demonstrated the involvement of the BDNF/TrkB pathway in tumor progression in gastric cancer [26].

With regard to the BDNF/TrkB signaling in CRC, BDNF or TrkB was overexpressed in both clinical tumor samples and associated with aggressive tumor phenotypes [27]–[30]. *In vitro* studies showed that BDNF/TrkB signaling was involved in proliferative or invasive properties [27]–[30], and efficacy or resistance of anti-epidermal growth factor receptor monoclonal antibody cetuximab [31] or gastrin-releasing peptide receptor [32]. These lines of evidence indicated that BDNF/TrkB signaling promotes tumor progression, leading to a poor prognosis for various human malignancies, and that it has emerged as a potential therapeutic target [33], [34].

The aim of this study was to examine: the association between BDNF/TrkB expression and clinicopathological variables in a series of human CRC tissues; the prognostic value of BDNF/TrkB signaling in CRC patients; and its therapeutic potential in vitro and in vivo.

## Materials and Methods

### Ethics Statement

This study was reviewed and approved by the Institutional Review Board and the Local Ethics Committee of the Mie University Graduate School of Medicine (No. 2126). Written informed consent was obtained from all the patients (adults and parents of children) enrolled onto the study.

The experimental protocols of in vivo studies were reviewed and approved by the Animal Care and Use Committee at the Mie University Graduate School of Medicine.

### Patients

A total of 223 patients with CRC, who were treated at the Department of Gastrointestinal and Pediatric Surgery in the Mie University Graduate School of Medicine from 2000 to 2008, were included in this study. Patients with incomplete clinical data, inadequate follow-up or inadequate tissue sampling were excluded from the study. All patients had histologically confirmed adenocarcinoma of the colon or rectum. The median age of the patients was 67 years (range: 12–91 years). The median follow-up time was 30.6 months (range: 1.5–107.2). A total of 49 patients died of CRC-related causes during this period.

Staging was based on clinical assessment and histopathological analysis using the International Union Against Cancer (UICC) TNM staging system. Written informed consent was obtained from all the patients enrolled onto the study, according to local ethics guidelines. This study was approved by the Institutional Review Board.

### Sample collections

Cancer tissues were frozen in liquid nitrogen immediately after surgical resection and stored at −80°C until use. The diagnosis of CRC was confirmed for all 223 patients based on clinicopathologic findings.

### Total RNA extraction, cDNA synthesis

Cancer tissues were minced and homogenized with a Mixer Mill MM 300 homogenizer (Qiagen, Chatsworth, CA, USA). Total RNA was isolated using an RNeasy mini kit (Qiagen) according to the manufacturer's instructions. cDNA was synthesized from 5 µg total RNA with a random hexamer primer and Superscript III reverse transcriptase (Invitrogen, Carlsbad, CA, USA) according to the manufacturer's instructions.

### Real-time quantitative reverse transcription polymerase chain reaction (RT-PCR) and relative gene expression levels

Quantitative PCR (qPCR) analysis was performed using a TaqMan Universal PCR Master Mix (Applied Biosystems, Foster City, CA, USA). The expression levels of the target gene transcripts, measured using TaqMan probes for BDNF (Assay ID, Hs00380947_m1) and TrkB (Assay ID, Hs00178811_m1), were normalized to GAPDH (Assay ID, Hs02758991_g1) and evaluated using Applied Biosystems StepOne Software (v2.1).

The relative BDNF and TrkB gene expression levels were determined using a standard curve. The standard curve was generated using a 5-fold serial dilution of random-primed qPCR Human Reference cDNA (TAKARA BIO INC., Clontech, Japan). All standard curves were linear within the range used for analysis, with an acceptable corresponding correlation coefficient (R^2^). The level of expression of the target gene was calculated from the standard curve, and normalized against GAPDH. Finally, the target gene mRNA level was expressed as a ratio relative to the GAPDH mRNA level. Real-time quantitative PCR assays were performed in triplicate for each sample and the mean value was used in calculating mRNA expression levels.

Amplified PCR products were separated electrophoretically, visualized, and photographed under UV light after ethidium bromide staining.

### Immunohistochemistry

Immunohistochemistry was performed as described previously [25]. Sections 2–3 µm thick were cut from the formalin-fixed paraffin-embedded (FFPE) specimens of the CRC patients. After deparaffinization and dehydration, the specimens were boiled in 10 mM sodium citrate buffer for 15 min to unmask the antigens. Sections were then blocked with normal goat serum (Vector Laboratories Inc., Burlingame, CA, USA) for 60 min and incubated with primary antibody overnight at 4 °C. Antibody binding was detected using Envision reagents (Envision kit/HRP, Dako Cytomation, Denmark). All sections were counterstained with hematoxylin. A primary rabbit polyclonal antibody against BDNF (H-117, 1∶350; Santa Cruz Biotechnology, Santa Cruz, CA, USA) and a primary mouse monoclonal antibody against TrkB (1∶100; R&D Systems, Foster City, CA, USA) were used. Negative controls were also run simultaneously by the exclusion of the respective primary antibody.

### CRC cell lines

Human colorectal cancer cell lines DLD1, LoVo, SW480, HT29, and CaCO2 were obtained from the Cell Resource Center for Biomedical Research (Tohoku University, Japan). The cell line authentication testing has been performed for these cell lines. These cell lines were maintained in RPMI-1640 medium supplemented with 10% fetal bovine serum, 100 IU/mL penicillin, and 100 µg/mL streptomycin at 37 °C and 5% CO2.

### Reagents

Recombinant human BDNF was purchased from PeproTech (Rocky Hill, NJ, USA) and prepared according to the manufacturer's instructions. Recombinant human BDNF was dissolved in PBS (10 µg/ml) for the *in vitro* assays.

K252a was purchased from Calbiochem (San Diego, CA, USA) and stored at −20°C before use. K252a was dissolved in PBS (10 µg/ml) for the *in vitro* and *in vivo* assays.

### Western blot analysis

The CRC cell lines were washed in ice-cold PBS whilst on the dish. Cold lysis buffer (Tris-buffered saline, pH 7.5, containing 1% Triton X-100) was then added directly to the dish. The cells were then scraped off the dish, collected, and homogenized using a Mixer Mill MM 300 homogenizer (Qiagen Inc., Chatsworth, CA, USA). The supernatants were collected and frozen at −20°C until use. The protein concentration was measured using the BCA protein assay (Pierce, Rockford, IL, USA). 20 µg of the protein lysate was mixed with an equal volume of 2× Laemmli loading buffer containing 2-mercaptoethanol, and heated at 100 °C for 5 min. Samples were electrophoretically separated on 12.5% gradient polyacrylamide gels containing 0.1% SDS, followed by semi-dry transfer to an Immun-Blot PVDF membrane (Bio-Rad Laboratories, Hercules, CA, USA). The membrane was then blocked using 5% skimmed milk in Tris-buffered saline, pH 7.5, supplemented with 0.1% Tween 20 (TBS-T).

The blots were then incubated with mouse monoclonal anti-TrkB antibody (R&D Systems, Foster City, CA, USA) at a 1∶1000 dilution, rabbit polyclonal anti-BDNF antibody (H-117, 1∶350; Santa Cruz Biotechnology, Santa Cruz, CA, USA) at a 1∶100 dilution, and mouse monoclonal anti-actin (clone C4) antibody (MP Biomedicals, LLC, Solon, OH, USA) at a 1∶400 dilution in 5% skimmed milk in TBS-T overnight at 4 °C. After washing three times in TBS-T, the blots were incubated with alkaline-phosphatase-conjugated goat anti- mouse IgG (Promega, Madison, WI, USA) at a 1∶200 dilution in 5% skimmed milk in TBS-T. Following treatment with an enhanced chemiluminescence detection solution, chemiluminescent signals were visualized in a CS Analyzer and AE-6962 light capture (ATTO Corp., Tokyo, Japan).

### Cell viability assay

To assess the effect of BDNF on cell viability, migration, invasion, and anoikis resistance, recombinant human BDNF and K252a, a selective pharmacological pan-Trk inhibitor, were used.

Cell viability, migration, invasion, and anoikis resistance were compared among non-treated cells (control), BDNF-treated cells, K252a-treated cells, and K252a followed by BDNF-treated cells.

Cytotoxicity was evaluated using a WST-8 [2-(2-methoxy-4-nitrophenyl)-3-(4-nitrophenyl)-5-(2, 4-disulfophenyl)-2H-tetrazolium, monosodium salt] colorimetric assay. Cancer cells (5000 cells/well) were seeded onto 96-well cell plates (Becton Dickinson Labware, Franklin Lakes, NJ, USA) in 100 µl of culture medium for 24 h. After preincubation, the cells were treated with K252a (100 nM) or serum-free medium for 2 h, and then treated with either recombinant human BDNF (100 ng/ml) or serum-free medium. 48 h later, the medium was discarded and replaced with 90 µl of fresh medium followed by the addition of 10 µl WST-8 reagent solution (Cell Counting Kit, DOJINDO LABORATORIES, Japan) and incubated for 2 h at 37 °C in an incubator.

Cell viability was determined by colorimetric comparison by reading optical density (OD) values from a microplate reader (SoftMax, Molecular Devices Corporation, CA, USA) at an absorption wavelength of 450 nm. Cytotoxicity was evaluated using the Cell Counting Kit according to the manufacturer's instructions. The data were obtained from similar results of at least three independent experiments. Results are presented as mean ± standard error (SE).

### Migration assay

Confluent cancer cells were serum-deprived for 48 h. Wounds were generated using a sterile 200-µL pipette tip after preincubation with K252a (100 nM) or serum-free medium for 2 h. Recombinant human BDNF (100 ng/ml) or serum-free medium was added and the cells were incubated at 37 °C for an additional 46 h. Wound closure was assessed using an Olympus IX71 microscope (Olympus, Center Valley, PA, USA) at 10× magnification. Cell migration distance was measured using Adobe Photoshop 9.0.2 software and compared with baseline measurements. Each independent experiment was performed at least three times. Results are presented as mean ±SE.

### Invasion assay

Cell invasion was evaluated using Biocoat Matrigel invasion chambers and control inserts (Becton Dickinson Labware). A total of 50000 cells/well were seeded in the invasion and control chambers, and pre-incubated with 100 nM K252a or serum-free medium for 2 h. Fresh medium alone or medium containing recombinant human BDNF (100 ng/ml) was then added to the Falcon companion plates (BD Biosciences, San Jose, CA). The Matrigel invasion chambers and control inserts were incubated for 24 h at 37 °C. The incubation medium containing cells was removed from the top chamber using cotton swabs and serum-free medium. The membranes were fixed in methanol, stained with Mayer's hematoxylin, dehydrated in ethanol, and mounted on glass slides. The number of cells that invaded the underside of the membrane was then determined. Each independent experiment was performed three times. Results are presented as mean ±SE.

### Anoikis assay

Anoikis, detachment-induced apoptosis, is known to be induced when adherent tumor cells are forced to grow in a non-adherent fashion. Anoikis resistance was evaluated by the proportion of viable tumor cells that proliferated non-adherently in low-attachment dishes.

Anoikis assays were performed in six-well Costar Ultra Low Attachment Microplates (Corning, NY, USA). DLD1, LoVo, and SW480 cells were suspended in RPMI-1640 with BDNF (100 ng/ml), K252a (100 nM), or K252a+BDNF at a concentration of 5×10^5^ cells/ml, respectively. Their cell suspensions (2 ml) were added to each well and incubated for 24 h in a humidified atmosphere (37 °C and 5% CO_2_).

After induction of anoikis, cells were seeded at 5×10^3^ cells/well in microtiter plates (96 wells, flat bottom) in a final volume of 100 µL culture medium per well. To assess the viable tumor cells that proliferated non-adherently in low-attachment dishes, spectrophotometric absorbance of each well was measured using WST-8 reagent solution as described above (cell viability assay). Each independent experiment was performed six times. Results are presented as mean ±SE.

After induction of anoikis, 5×10^5^ cells were washed and resuspended in 0.5 ml of 1×binding buffer, and Annexin V: fluorescein isothiocyanate/Propidium Iodide (PI) labeling performed according to the manufacturer's protocol (Bio Vision, Mountain View, CA, USA). The analysis was performed using a FACSCalibur flow cytometer (BD Biosciences, San Jose, CA) to quantify the proportion of viable or apoptotic tumor cells under non-adherent growth condition using low-attachment dishes. Each sample contained 5×10^5^ cells. The data were analyzed using CellQuest Pro software (BD Biosciences). Viable tumor cells that were negative for both Annexin V and PI staining (lower left quadrant) were considered as anoikis-resistant cells with non-adherent growth. Apoptotic tumor cells that were positive for Annexin V staining (lower right quadrant+upper right quadrant) were considered as anoikis-induced cells. Tumor cells treated with 2.5% formalin solution were used as a positive control for apoptosis induced cells. Flow cytometric analysis was performed to confirm the results of cell viability assay for anoikis resistance.

### *In vivo* peritoneal metastasis assay

Male nude mice (BALB/c) at 8 weeks of age were purchased from Japan SLC Inc. (Shizuoka, Japan). The experimental protocols were reviewed and approved by the Animal Care and Use Committee at the Mie University Graduate School of Medicine. DLD1 cells (5×10^7^ cells/500 µl PBS) were injected intraperitoneally for peritoneal metastatic formation. Either K252a (500 µg/kg) or PBS (control) was injected intraperitoneally three times a week to examine the effect of K252a on the peritoneal metastatic formation. Four weeks later, the mice were sacrificed, and then the size and number of peritoneal metastatic nodules was evaluated (each group; n = 5). Results are presented as mean ±SE.

### Statistical analysis

All statistical analyses were performed using JMP version 5 (SAS Institute Inc. Cary, NC, USA). The results are expressed as the mean ±SE (standard error). Differences between groups were estimated using the Pearson's chi-square test, repeated-measures analysis of variance (ANOVA) analysis, or an unpaired Student's t-test. Actuarial survival curves were obtained using the Kaplan–Meier method, and comparisons were made using log-rank tests. Univariate and multivariate analyses were done using the Cox proportional hazards model to investigate the effects of the histopathological and molecular factors (BDNF and TrkB expression status) present in the primary tumor specimen on overall survival (OS). Variables associated with survival with a P-value less than 0.05 in univariate analysis were used for multivariate analysis. P-values of less than 0.05 were considered statistically significant. Two-sided P-values <0.05 were considered to be statistically significant.

## Results

### The BDNF mRNA level in CRC tissues

The BDNF mRNA expression ratio, relative to GAPDH, in CRC tissues was 0.122±0.230 (mean±SD), ranging from 0 to 1.357. For statistical analysis purposes the BDNF mRNA expression values were dichotomized as low or high. A cutoff value for the BDNF mRNA level was determined as a maximum predictive value using an OS based on receiver–operating characteristic (ROC) curve analysis. One hundred and two patients had high BDNF mRNA expression (>0.037), whereas 121 patients had low BDNF mRNA expression. As shown in Table 1, the BDNF mRNA expression level in CRC tissues was found to be significantly associated with synchronous liver metastasis (P = 0.007) and synchronous peritoneal metastasis (P = 0.026). No significant association was observed between the BDNF mRNA in CRC tissues and any other clinicopathological variables.

**Table 1:** Association of BDNF, TrkB, and co-expression of BDNF/TrkB with clinicopathological variables.

Variables		BDNF mRNA			TrkB mRNA			Co-expression		
		expression			expression			of BDNF/TrkB		
		high	low		high	low		high	low	
	n	(n = 102)	(n = 121)	*p*	(n = 171)	(n = 52)	*p*	(n = 94)	(n = 129)	*p*
**Gender**										
male	129	62	67	0.415	99	30	0.979	58	71	0.32
female	94	40	54		72	22		36	58	
**Age (year)**										
<67 (median)	99	51	48	0.122	76	23	0.978	47	52	0.151
≧67	124	51	73		95	29		47	77	
**Tumor Location**										
colon	153	73	80	0.382	118	35	0.817	69	84	0.188
rectum	70	29	41		53	17		25	45	
**Tumor Size (cm)**										
≧4.5 cm (median)	106	49	57	0.889	85	21	0.239	46	60	0.72
<4.5 cm	117	53	64		86	31		48	69	
**Histological type**										
differentiated	202	89	113	0.118	154	48	0.627	82	120	0.144
undifferentiated	21	13	8		17	4		12	9	
**Blood vessel**										
**involvement**										
present	170	79	91	0.695	131	39	0.811	74	96	0.456
absent	53	23	30		40	13		20	33	
**Lymphatic vessel**										
**involvement**										
present	199	93	106	0.391	153	46	0.837	86	113	0.354
absent	24	9	15		18	6		8	16	
**Serosal invasion**										
present	53	25	28	0.811	43	10	0.38	24	29	0.597
absent	170	77	93		128	42		70	100	
**Lymph node metastasis**										
present	128	52	76	0.075	91	37	0.022*	48	80	0.102
absent	95	50	45		80	15		46	49	
**Liver metastasis**										
present	183	76	107	0.007*	137	46	0.17	71	112	0.03*
absent	40	26	14		34	6		23	17	
**Peritoneal carcinomatosis**										
present	213	94	119	0.026*	161	52	0.074	86	127	0.013*
absent	10	8	2		10	0		8	2	
					Pearson's chi-square test			*P<0.05		

TrkB mRNA expression indicates mRNA transcript levels of both TrkB.FL and TrkB.T1.

### The TrkB mRNA level in CRC tissues

According to the TaqMan Gene Expression Assay (Applied Biosystems, Foster City, CA, USA), probe of TrkB (Assay ID, Hs00178811_m1) is designed to span the exon 6/7 boundaries. Corresponding primers amplify the fragments containing exons 6–7 which encodes the extracellular portion of the TrkB receptor. Therefore, this primer and probe set detects both the full-length TrkB (TrkB.FL) and the truncated TrkB (TrkB.T1) [35].

The TrkB mRNA expression ratio, relative to GAPDH, in CRC tissues was 0.054±0.141 (mean±SD), ranging from 0 to 1.149. ROC curve analysis showed that the cutoff value for TrkB mRNA was 0.002 (a maximum predictive value for OS). One hundred and seventy-one patients had high TrkB mRNA expression, whereas 52 patients had low TrkB mRNA expression. As shown in Table 1, the TrkB mRNA level in the CRC tissues was found to be significantly associated with lymph node metastasis (P = 0.022). No significant association was observed between the TrkB mRNA level in CRC tissues and any other clinicopathological variables.

### Co-expression of BDNF and TrkB in CRC tissues

Ninety-four patients showed both high BDNF and TrkB mRNA expression. This patient group was classified as a co-expressing both BDNF and TrkB. As shown in Table 1, the co-expression of BDNF and TrkB in CRC tissues was found to be significantly associated with synchronous liver metastasis (P = 0.03) and synchronous peritoneal metastasis (P = 0.013). No significant association was observed between the TrkB mRNA level in the CRC tissues and any other clinicopathological variables.

### The prognostic impact of BDNF, TrkB, and the co-expression of BDNF and TrkB in colorectal cancer tissues

**Figure 1:**
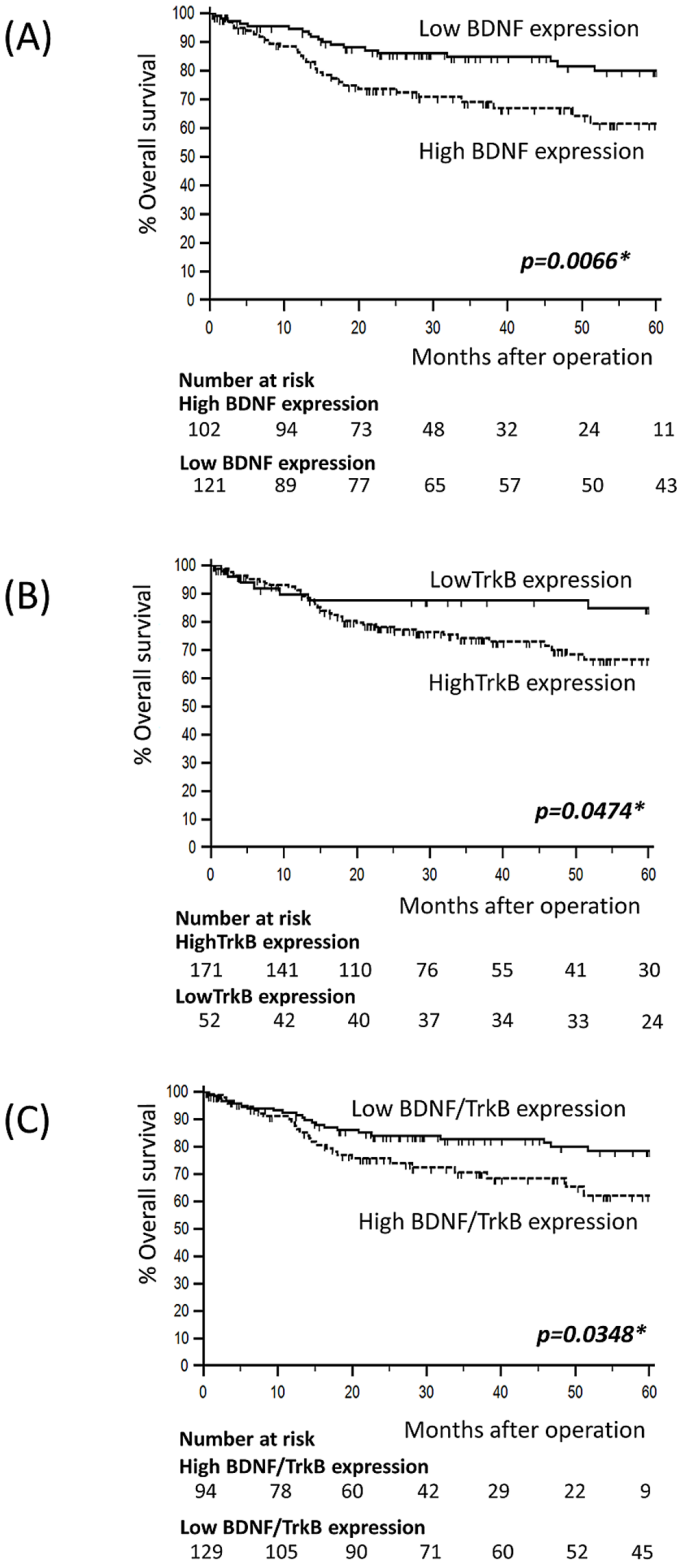
Kaplan-Meier plots of overall survival according to BDNF, TrkB, and co-expression of BDNF/TrkB. (A): Comparison between the overall survival of patient groups with high BDNF mRNA expression (n = 102) and those with low BDNF mRNA expression (n = 121). (B): Comparison between the overall survival of patient groups with high TrkB mRNA expression (n = 171) and those with low TrkB mRNA expression (n = 52). (C): Comparison between the overall survival of patients groups with high co-expression of BDNF and TrkB (n = 94) and those without it (n = 129). TrkB mRNA expression indicates mRNA transcript levels of both TrkB.FL and TrkB.T1.

Figure 1 shows Kaplan-Meier curves for BDNF (A), TrkB (B), and the co-expression of BDNF and TrkB (C), respectively. CRC patients with high BDNF mRNA expression (n = 102) had a significantly worse OS than those with low BDNF mRNA expression (n = 121, log-rank test, P = 0.0066). Similarly, CRC patients with high TrkB mRNA expression (n = 171) had a significantly worse OS than those with low TrkB mRNA expression (n = 52, log-rank test, P = 0.0474). CRC patients whose tumors co-expressed BDNF and TrkB (n = 94) had a significantly worse OS than those with the other expression pattern (n = 129, log-rank test, P = 0.0348).

### BDNF and TrkB protein expression in the primary and metastatic tumors

**Figure 2:**
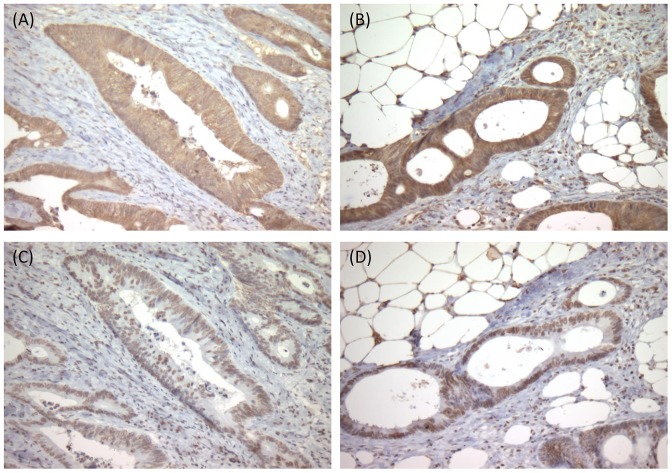
BDNF and TrkB protein expression in primary and metastatic colorectal cancer. Representative images of immuno-reactive BDNF and TrkB protein expression in primary CRC and peritoneal metastasis are shown (original magnification: 100×). Anti TrkB antibody (R&D Systems, Foster City, CA, USA) detected both TrkB.FL and TrkB.T1 proteins, which was confirmed by Western blotting analysis. (A): The immunoreactive BDNF protein is located in the cytoplasm of the tumor cells of the primary CRC. (B): The immunoreactive BDNF protein is located in the cytoplasm of the tumor cells in the corresponding peritoneal metastasis. (C): The immunoreactive TrkB protein is located in the nucleus of the tumor cells of the primary CRC. (D): The immunoreactive TrkB protein is located in the nucleus of the tumor cells in the corresponding peritoneal metastasis. These expression patterns for the BDNF and TrkB proteins were confirmed in CRC patients (n = 5) whose primary and peritoneal metastatic nodules were available for immunohistochemistry.

Immunohistochemistry showed that the BDNF protein (A, B) was expressed in the cytoplasm of human CRC cells and that the TrkB protein (C, D) was expressed in the nucleus of human CRC cells (Figure 2). In patients with both primary tumor (A, C) and peritoneal metastatic nodules (B, D), human CRC cells at the peritoneal metastatic nodules expressed both BDNF and TrkB, as did those at the primary tumor. We confirmed that anti TrkB antibody (R&D Systems, Foster City, CA, USA) detected both TrkB.FL and TrkB.T1 based on the results of Western blotting analysis using human CRC cell lines (data shown in the following section).

### Expression of BDNF and TrkB in CRC cells in vitro

As shown by the above results, both BDNF and TrkB expression in tumor cells seems to be involved in both primary and metastatic tumor progression in human CRC. To explore the involvement of an autocrine BDNF/TrkB signaling in CRC progression, we first examined BDNF and TrkB expression in 5 CRC cell lines including CaCO2, DLD1, HT29, LoVo, and SW480.

**Figure 3:**
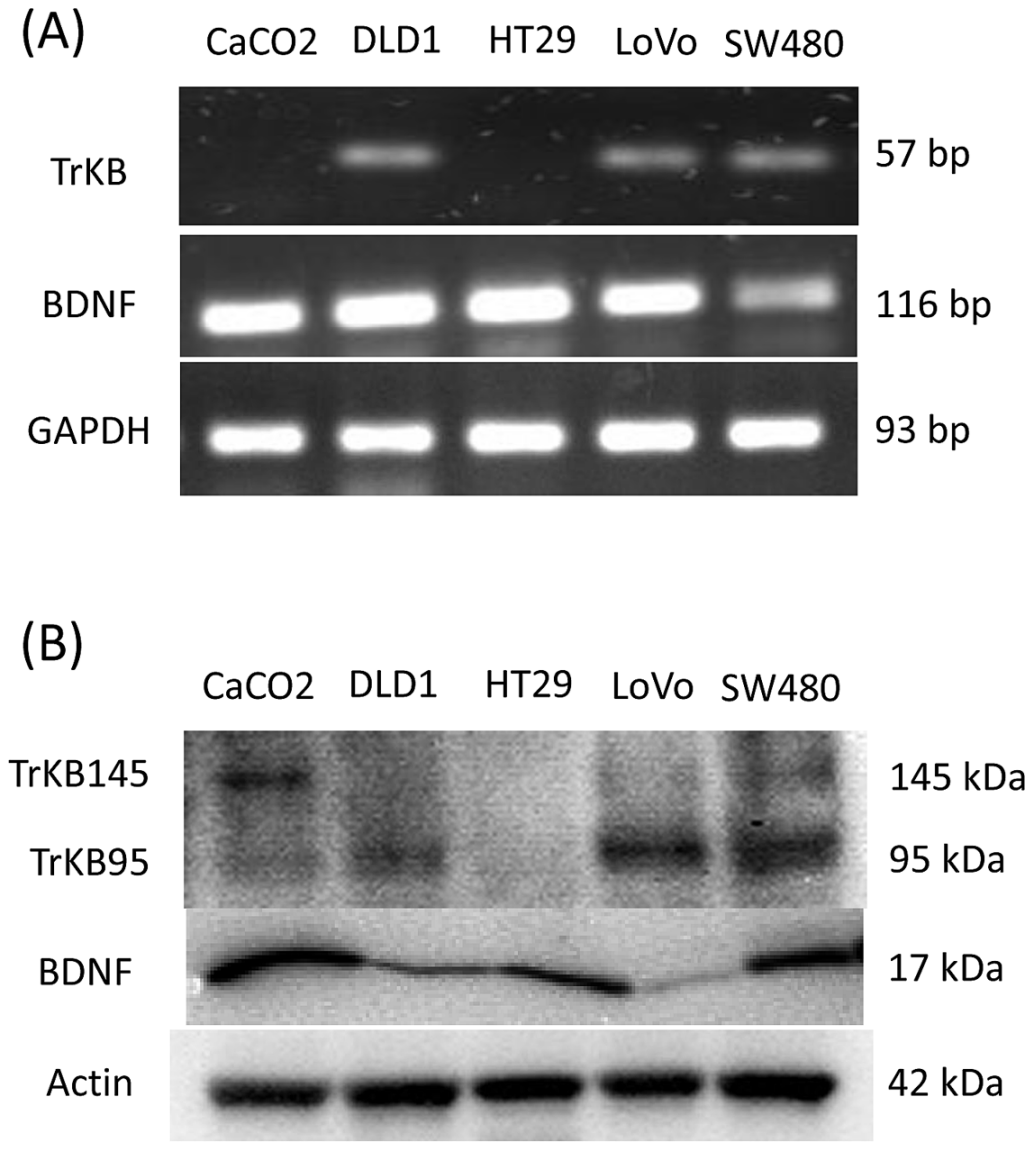
Expression of BDNF and TrkB in CRC cells in vitro. (A): RT-PCR analysis of BDNF and TrkB mRNA levels in CRC cell lines CaCO2 (lane: 1), DLD1 (lane: 2), HT29 (lane: 3), LoVo (lane: 4), and SW480 (lane: 5). GAPDH mRNA levels was used as an internal control. TrkB mRNA levels contain both TrkB.FL and TrkB.T1 mRNAs. (B): Western blotting analysis of BDNF and TrkB protein levels in CRC cell lines CaCO2 (lane: 1), DLD1 (lane: 2), HT29 (lane: 3), LoVo (lane: 4), and SW480 (lane: 5). The full-length TrkB (TrkB.FL, 145 kDa) and the truncated TrkB (TrkB.T1, 95 kDa) were detected. Actin was used as an internal control.

BDNF mRNA expression was detected in all 5 cell lines by RT-PCR analysis (Figure 3A). In contrast, TrkB mRNA expression (containing both TrkB.FL and TrkB.T1) was detected in 3 out of 5 cell lines (DLD1, LoVo, and SW480).

Western blotting analysis (Figure 3B) showed that TrkB.FL (145 kDa) was detected in 3 cell lines (CaCO2, LoVo, and SW480) and that TrkB.T1 (95 kDa) was also detected in 4 cell lines (CaCO2, DLD1, LoVo, and SW480). HT29 have neither TrkB.FL nor TrkB.T1 protein. BDNF protein was detected in all 5 cell lines as well as mRNA expression.

Based on our results and evidence from previous reports, we selected 3 cell lines (DLD1, LoVo, and SW480) which showed both BDNF and TrkB mRNA expression with either TrkB.FL or TrkB.T1 protein expression for further examination.

### BDNF increases cell viability of TrkB-expressing CRC cells

**Figure 4:**
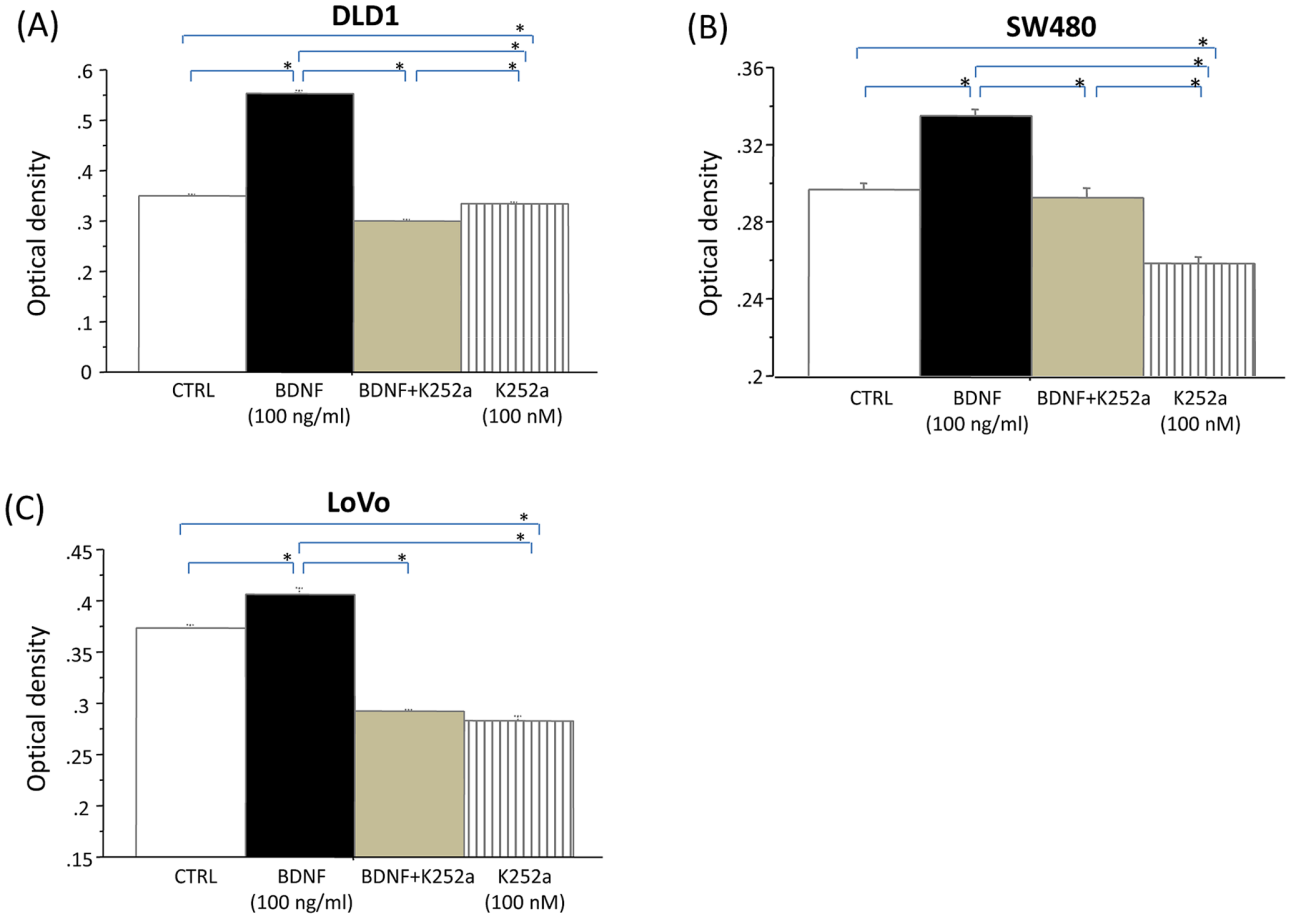
BDNF increases cell viability in TrkB-expressing CRC cells. BDNF/TrkB-expressing DLD1 (A), SW480 (B), and LoVo (C) cells were used to examine the effect of BDNF, K252a, and their combination on tumor cell viability. Each DLD1, LoVo, and SW480 cell was treated with BDNF (100 ng/ml), K252a (100 nM), or K252a followed by BDNF for 48 h. Exogenous BDNF increased tumor cell viability in TrkB-expressing CRC cells, and K252a inhibited tumor cell viability. The data were obtained from similar results of at least three independent experiments. Results are presented as mean ±SE. *; P<0.05.

To explore the possibility of an autocrine BDNF/TrkB signaling loop occurring in CRC, we investigated the effect of BDNF on tumor cell viability in TrkB-expressing CRC cells. As shown in Figure 4, DLD1 (A), SW480 (B), and LoVo (C) cells were each treated with BDNF (100 ng/ml), K252a (100 nM), or K252a followed by BDNF for 48 h. BDNF significantly increased the viability of these cell lines, as compared with non-treated controls. K252a significantly decreased the viability of these cell lines as compared with the non-treated controls. K252a followed by BDNF inhibited cell viability of these cell lines, as compared with BDNF treatment or non-treated controls.

These results suggest that exogenous BDNF increases tumor cell viability in TrkB-expressing CRC cells, and that TrkB receptor blockade may provide a potent means of inhibiting tumor growth.

### BDNF promotes migration in TrkB-expressing CRC cells

**Figure 5:**
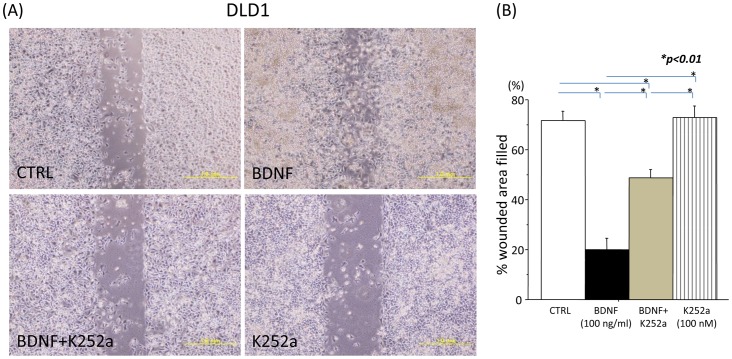
BDNF promotes migration in TrkB-expressing CRC cells. (A): Representative images from migration assays for DLD1 cells are shown (original magnification; 100×). Tumor cells were treated with medium (control), BDNF (100 ng/ml), K252a (100 nM), or K252a followed by BDNF. (B): The bar graph of these results indicates that exogenous BDNF enhanced tumor cell motility in TrkB-expressing CRC cells, and that K252a inhibited the migratory ability of these tumor cells. The data were obtained from similar results of at least three independent experiments. Results are presented as mean ±SE. *; P<0.01.

To examine the effect of BDNF on tumor cell motility, DLD1 cells were used for *in vitro* migration assays. Forty-eight hours later, BDNF significantly increased the migration of DLD1 cells, as compared with the non-treated controls. K252a decreased the migratory ability of the DLD1 cells, which was nearly identical to the non-treated controls. K252a followed by BDNF inhibited the migratory ability of DLD1 cells, as compared with BDNF treatment. The representative images of the above results were shown in Figure 5A. Quantitative analysis of cell migration was also shown (Figure 5B). These results suggest that exogenous BDNF enhances tumor cell motility in TrkB-expressing CRC cells, and that TrkB receptor blockade may potently inhibit the migratory ability of these tumor cells.

### BDNF promotes invasion in TrkB-expressing CRC cells

**Figure 6:**
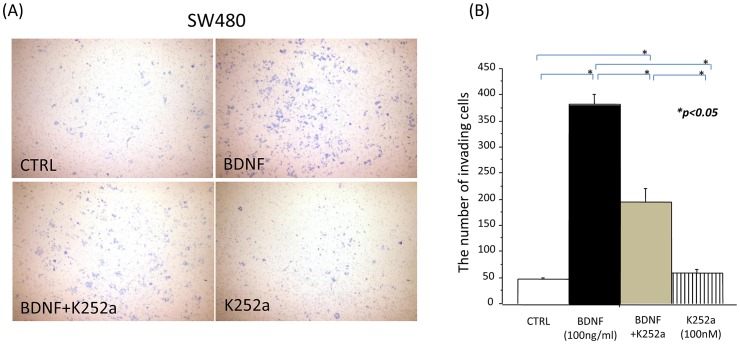
BDNF promotes invasion in TrkB-expressing CRC cells. (A): Representative images of an invasion assay with SW480 cells are shown (original magnification; 100×). Tumor cells were treated with medium (control), BDNF (100 ng/ml), K252a (100 nM), or K252a followed by BDNF. (B): The bar graph of these results indicates that exogenous BDNF enhanced tumor cell invasion in TrkB-expressing CRC cells, and that K252a inhibited the invasive ability of these tumor cells. The data were obtained from similar results of at least three independent experiments. Results are presented as mean ±SE. *; P<0.05.

To examine the effect of BDNF on tumor cell invasion, SW480 cells were used for *in vitro* invasion assays. Twenty-four hours later, BDNF significantly increased the invasion of SW480 cells, as compared with the non-treated controls. K252a decreased the invasive ability of SW480 cells, which was nearly identical to the non-treated controls. K252a followed by BDNF treatment inhibited the invasive ability of SW480 cells, as compared with BDNF treatment. The representative images of the above results were shown in Figure 6A. Quantitative analysis of invading tumor cells was also shown (Figure 6B). These results suggest that exogenous BDNF enhances tumor cell invasion in TrkB-expressing CRC cells, and that a TrkB receptor blockade may potently inhibit the invasive ability of these tumor cells.

### BDNF enhances anoikis resistance in TrkB-expressing CRC cells

**Figure 7:**
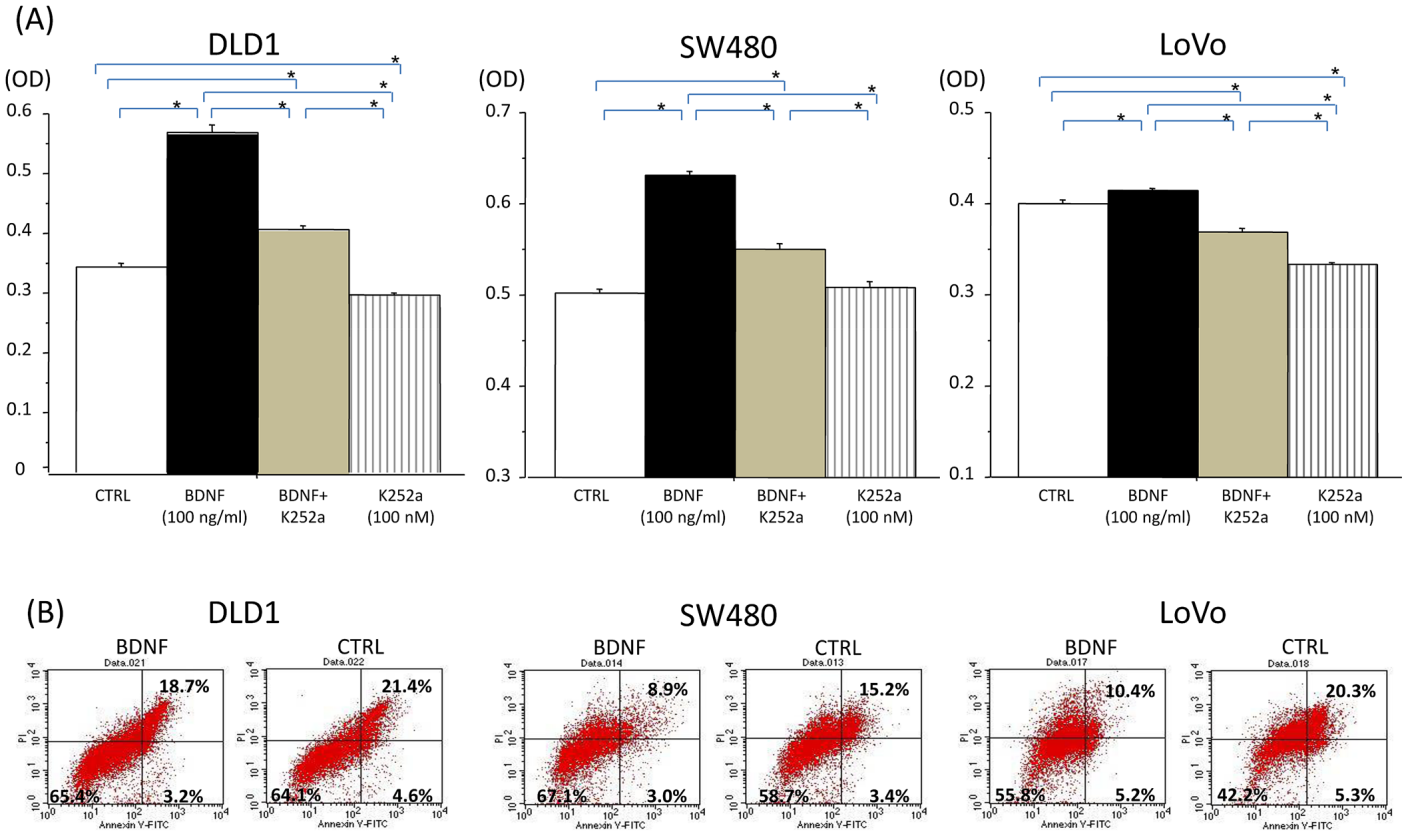
BDNF enhances anoikis resistance in TrkB-expressing CRC cells. The upper panel (A) shows the number of anoikis resistant cells by counting viable tumor cells that proliferated non-adherently in low-attachment dishes using a WST-8 reagent (cell viability assay). The lower panel (B) shows the representative flow cytometric data showing the proportion of viable tumor cells (lower left quadrant) and apoptotic tumor cells (lower right quadrant+upper right quadrant). DLD1, SW480, and LoVo cells were treated with medium (control), BDNF (100 ng/ml), K252a (100 nM), or K252a followed by BDNF. The floating tumor cells were subjected to cell viability assay and flow cytometric analysis. Exogenous BDNF enhances anoikis resistance in TrkB-expressing CRC cells (increased viable tumor cells), and K252a inhibits the anoikis resistance of tumor cells. Quantitative analysis was performed for cell viability assay. Each independent experiment was performed six times. Results are presented as mean ±SE. *; P<0.05.

Anoikis is a form of detachment-induced apoptosis. In the *in vitro* experimental setting, it is induced when adherent tumor cells are forced into non-adherent growth. To examine the effect of BDNF on anoikis resistance (detachment-induced apoptosis resistance), DLD1, LoVo, and SW480 cells were used for *in vitro* anoikis assays. The number of anoikis resistant cells was evaluated by counting viable tumor cells that proliferated non-adherently in low-attachment dishes (cell viability assay using a WST-8 reagent solution). As shown in Figure 7A, BDNF (100 ng/ml) significantly increased the number of anoikis-resistant tumor cells (floating viable tumor cells with non-adherent growth), as compared with non-treated controls. K252a (100 nM) decreased the number of anoikis-resistant tumor cells as compared with the non-treated control. K252a followed by BDNF treatment inhibited the anoikis resistance of tumor cells, as compared with BDNF treatment. The representative data of flow cytometric analysis after anoikis indiction assay were shown in Figure 7B. BDNF increased the proportion of viable tumor cells (lower left quadrant), as compared with non-treated controls (DLD1; 64.1%→65.4%, SW480; 58.7%→67.1%, LoVo; 42.2%→55.8%). The proportion of apoptotic tumor cells (lower right quadrant+upper right quadrant) was decreased by BDNF (DLD1; 26.0%→21.9%, SW480; 18.6%→11.9%, LoVo; 25.6%→15.6%), suggesting an anti-apoptotic effect by BDNF.

These results suggest that exogenous BDNF enhances anoikis resistance in TrkB-expressing CRC cells, and that a TrkB receptor blockade may inhibit the anoikis resistance of these tumor cells.

### K252a suppresses the peritoneal metastasis of BDNF/TrkB-co-expressing CRC cells *in vivo*

**Figure 8:**
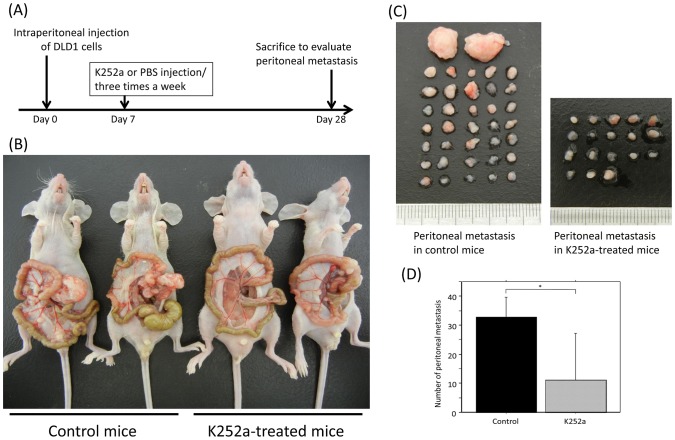
K252a suppresses the peritoneal metastasis of BDNF/TrkB-co-expressing CRC cells *in vivo*. (A): The experimental schedule of the *in vivo* peritoneal metastasis assay. DLD1 cells (5×10^7^ cells/500 µl PBS) were injected intraperitoneally (day 0). Seven days later (day 7), K252a (500 µg/kg) or PBS (control) was injected three times a week. Four weeks later (day 28), mice were sacrificed, and then the size and number of peritoneal metastatic nodules were evaluated (for each group; n  =  5). (B): Representative images of control mice and K252a-treated mice. (C): Representative images of peritoneal metastases in control mice and K252a-treated mice. (D): The bar graph indicates that the amount of peritoneal metastasis was significantly lower in K252a-treated mice than in control mice. Each value represents the mean ±SE. *; P<0.05.

The results presented above have shown that BDNF enhances the cell viability, migration, and anoikis resistance of TrkB-expressing CRC cells, and that the TrkB inhibitor, K252a, inhibited these effects. These results suggest that an autocrine BDNF/TrkB loop may be involved in the progression of both primary and peritoneal metastatic tumors. We next investigated whether a TrkB receptor blockade suppress the peritoneal metastasis of BDNF/TrkB-co-expressing DLD1 cells using an *in vivo* peritoneal metastasis assay. Figure 8A showed an experimental schedule of the *in vivo* peritoneal metastasis assay. The representative images of PC arising from DLD1 cells and peritoneal metastases were shown in Figure 8B and 8C, respectively. Quantitative analysis showed that the size and number of peritoneal metastatic nodules were significantly smaller and lower, respectively, in K252a-treated mice, as compared with control mice (Figure 8D). Results are presented as mean ±SE. *; P<0.05

These results suggest that a TrkB receptor blockade may inhibit either anoikis resistance, adhesion onto the peritoneal surface, or the colonization of the peritoneal cavity.

## Discussion

Previously, we reported an inverse correlation between TrkB and E-cadherin mRNA levels in CRC patients, and an inhibition of tumor cell proliferation, migration, and invasion by TrkB knockdown in CRC cell lines [25]. These results indicate that the overexpression of TrkB may induce EMT in CRC cells, resulting in tumor progression and poor survival. In this study, we focused on the association between a TrkB receptor ligand, BDNF, and TrkB (BDNF/TrkB signaling) in CRC to clarify its biological role and its therapeutic potential in CRC.

The mRNA levels of BDNF, TrkB and their co-expression in CRC tissues were associated with tumor progression and poor prognosis of CRC patients. Although we couldn't show that co-expression of BDNF and TrkB had an independent prognostic significance by multivariate analysis (data not shown), BDNF/TrkB expression status of the primary CRC tissues may provide prognostic information for CRC patients.

Previous reports showed that BDNF was higher in CRC tissues than adjacent normal tissues at the mRNA [30] or protein [27] level. Akil and colleagues also demonstrated that mRNA levels of BDNF, TrkB.FL and TrkB.T1 were higher in CRC tissues than in the paired adjacent normal tissues or non-CRC tissues (megadolicocolon) [28]. With regard to BDNF expression status of non-pathological colon, Lommatzsch and colleagues showed that BDNF mRNA was produced by colonic epithelial cells, several types of smooth muscle, and neurons of myenteric plexus [36]. Interestingly, colonic epithelial cells which expressed BDNF lacked its receptors, TrkB and p75NTR, suggesting that BDNF/TrkB autocrine loop doesn't exist in normal epithelial cells.

We found that BDNF/TrkB signaling was significantly associated with proliferation, migration, invasion, and anoikis resistance in BDNF/TrkB co-expressing CRC cells. Recent studies also demonstrated the molecular mechanisms of BDNF/TrkB signaling in tumor cell migration, invasion, and anoikis resistance. They showed the involvement of β5 integrin in BDNF/TrkB signaling mediated cell migration [37], BDNF/TrkB signaling induced invasion through matrix metalloproteinase-2 and -9 [38], and BDNF/TrkB signaling mediated EMT and anoikis resistance [39].

In this study, BDNF was detected at the mRNA and protein level in all 5 cell lines, although cell line-dependent difference was observed at the protein expression level. In contrast, TrkB was detected at the mRNA level in 3 out of 5 cell lines. Additionally, these 3 cell lines showed higher protein expression of TrkB.T1 than that of TrkB.FL.

TrkB.T1, one of the truncated forms of TrkB receptor, is generated by alternative splicing during gene transcription and lacks the intracellular tyrosine kinase domain with the extracellular BDNF binding region [35]. BDNF also binds TrkB.T1 as well as TrkB.FL. Although TrkB.T1 has long been regarded as negative regulator of BDNF/TrkB signaling [40], the functions of TrkB.T1 are still not clearly understood at present. Recent studies indicated that TrkB.T1 is a functional receptor regulating BDNF/TrkB.FL signaling [41] and mediating intracellular signaling related to cell growth and migration [42], [43].

TrkB.T1 was also frequently detected in several human malignancies rather than TrkB.FL [19], [44]. It was involved in invasion and metastasis of pancreatic cancer [44] or anti-apoptotic effect in breast cancer [19], suggesting a kinase-independent function in cancer.

In CRC cell lines, TrkB.T1 has been also reported to be preferentially expressed at both the mRNA and protein levels, as compared with TrkB.FL [28]. In these CRC cells, BDNF induced proliferation and anti-apoptotic effect were suppressed by pharmacological pan-Trk inhibitor, K252a [28]. These results are nearly consistent with our findings of *in vitro* studies.

Since TrkB.T1 regulates BDNF/TrkB.FL signaling [41] and mediates intracellular signaling related to cell growth and migration [42], [43], it should be clarified whether BDNF induced migration, invasion, and anoikis resistance are mediated by either a thyrosine kinase dependent function of BDNF/TrkB.FL or a kinase independent function of BDNF/TrkB.T1. Therefore, it is strongly necessary to evaluate the expression status of TrkB.FL and TrkB.T1. to examine the precise role of BDNF/TrkB signaling in cancer, although we didn't clarify it in this study.

We also demonstrated that treatment of mice with the pharmacological pan-Trk inhibitor K252a suppressed the PC formation of BDNF/TrkB-co-expressing CRC cells *in vivo*. PC arising from CRC has been reported to be found in approximately 7% of CRC patients at diagnosis, or at the time of primary tumor surgery (synchronous PC) [45]. Also, 4–12% of CRC patients developed PC after curative surgery (metachronous PC) [46]. Autopsy studies showed that PC was found in up to 40% of patients who die from CRC [47].

In our series, synchronous PC was the most powerful predictive factor of overall survival, with the highest risk ratio during the last 5 years of life (data not shown). These lines of evidence indicate that PC is regarded as a primary target of CRC treatment. Although the effects of chemotherapeutic treatment on the prognosis of metastatic CRC have been well reported, it remains unclear whether these treatments provide the same benefit to the subset of CRC patients with PC.

Recently, we showed that the blockade of BDNF/TrkB signaling by K252a suppressed the PC formation arising from gastric cancer cells with co-expression of BDNF and TrkB [26]. Our results of this study also indicated that BDNF/TrkB signaling may be a potential therapeutic target for PC arising from BDNF/TrkB-expressing CRC. BDNF/TrkB signaling may be a promising target for PC arising from gastrointestinal malignancies.

However, further studies will be needed to reveal the therapeutic potential of BDNF/TrkB signaling in CRC, especially for PC arising from CRC. To clarify the anti-tumor efficacy of the blockade of BDNF/TrkB signaling in CRC, we would first need to confirm whether a BDNF blockade, such as an anti-BDNF antibody, and a TrkB blockade, such as a pharmacological pan-Trk inhibitor or an antagonistic anti-TrkB antibody, show anti-tumor effects on CRC in patients with various BDNF/TrkB expression statuses.

In conclusion, we have demonstrated that BDNF/TrkB signaling in tumor tissues is associated with tumor progression and poor prognosis in CRC patients, and that it is also associated with proliferation, migration, invasion, and anoikis resistance in BDNF/TrkB co-expressing CRC cells. The BDNF and TrkB expression status in our resected specimens may provide prognostic information for CRC patients. Finally, BDNF/TrkB signaling may be a promising potential target for PC arising from CRC.
